# Dysconnectivity Within the Default Mode in First-Episode Schizophrenia: A Stochastic Dynamic Causal Modeling Study With Functional Magnetic Resonance Imaging

**DOI:** 10.1093/schbul/sbu080

**Published:** 2014-06-17

**Authors:** António J. Bastos-Leite, Gerard R. Ridgway, Celeste Silveira, Andreia Norton, Salomé Reis, Karl J. Friston

**Affiliations:** ^1^Department of Medical Imaging, Faculty of Medicine, University of Porto, Porto, Portugal;; ^2^Wellcome Trust Centre for Neuroimaging, Institute of Neurology, University College London, London, UK;; ^3^Department of Psychiatry, Hospital de São João, Porto, Portugal;; ^4^Department of Clinical Neurosciences and Mental Health, Faculty of Medicine, University of Porto, Porto, Portugal;; ^5^Hospital de Magalhães Lemos, Porto, Portugal; ^6^These authors contributed equally to the article.

**Keywords:** brain connectivity, default mode network, dysconnectivity, first-episode schizophrenia, functional magnetic resonance imaging (fMRI), resting state, stochastic dynamic causal modeling (DCM)

## Abstract

We report the first stochastic dynamic causal modeling (sDCM) study of effective connectivity within the default mode network (DMN) in schizophrenia. Thirty-three patients (9 women, mean age = 25.0 years, SD = 5) with a first episode of psychosis and diagnosis of schizophrenia—according to the Diagnostic and Statistic Manual of Mental Disorders, 4th edition, revised criteria—were studied. Fifteen healthy control subjects (4 women, mean age = 24.6 years, SD = 4) were included for comparison. All subjects underwent resting state functional magnetic resonance imaging (fMRI) interspersed with 2 periods of continuous picture viewing. The anterior frontal (AF), posterior cingulate (PC), and the left and right parietal nodes of the DMN were localized in an unbiased fashion using data from 16 independent healthy volunteers (using an identical fMRI protocol). We used sDCM to estimate directed connections between and within nodes of the DMN, which were subsequently compared with *t* tests at the between subject level. The excitatory effect of the PC node on the AF node and the inhibitory self-connection of the AF node were significantly weaker in patients (mean values = 0.013 and −0.048 Hz, SD = 0.09 and 0.05, respectively) relative to healthy subjects (mean values = 0.084 and −0.088 Hz, SD = 0.15 and 0.77, respectively; *P* < .05). In summary, sDCM revealed reduced effective connectivity to the AF node of the DMN—reflecting a reduced postsynaptic efficacy of prefrontal afferents—in patients with first-episode schizophrenia.

## Introduction

Schizophrenia is a complex psychiatric disorder of unknown etiology, with significant clinical and pathophysiological heterogeneity, for which biomarkers are still lacking.^1^ Schizophrenia is generally thought to result from pathological interactions among gray matter structures. In brief, there are 2 versions of this hypothesis. One is implied by Wernicke’s “sejunction” hypothesis, which postulated an anatomical disruption or “*dis*connection” of association fibers between regions. The other postulates abnormalities at the level of synaptic efficacy and plasticity, leading to “*dys*functional” integration or connectivity among cortical and subcortical systems.^2,3^ Neuroimaging studies of effective connectivity—defined as the causal influence of one neural system (eg, a network node) over another (or itself)—may, therefore, help to identify abnormalities in neural circuits whose dysfunction contributes to schizophrenia.

The default mode network (DMN) has been proposed as a system^4^ that may underlie introspective brain function and consciousness. Previous functional magnetic resonance imaging (fMRI) studies have demonstrated aberrant temporal correlations (functional connectivity) of the DMN in schizophrenia.^5,6^ It has been suggested that this abnormality could be attributable to an altered modulation of the anterior and posterior cingulate (PC) cortices or to result from abnormal interactions between those regions and other functional brain networks.^5^ In addition, differences between patients with schizophrenia and healthy control subjects have also been found with respect to functional connectivity among different brain networks.^7,8^

Dynamic causal modeling (DCM) is a Bayesian scheme for assessing effective connectivity. DCM uses the Bayesian inversion of neuronal network models that are grounded in neurophysiology and anatomy.^9^ The advantages of studying effective connectivity with DCM include the ability to compare different models of brain networks, as well as to characterize directed connectivity between cortical nodes at a neuronal level.^10^ Another advantage of DCM is that it entails an explicit model of neuronal coupling, which enables regional variations in hemodynamic parameters to be estimated. This precludes difficulties in the interpretation of functional connectivity among hemodynamic signals in the presence of hemodynamic variability.^11^

The particular advantage of stochastic DCM (sDCM) over the conventional (deterministic) DCM approach is that one can model endogenous fluctuations in neuronal activity. In other words, whereas deterministic DCM generates probabilistic and parametric measures of effective connectivity as a response to experimental exogenous inputs (eg, periods of “activity” during typical block or event-related fMRI experiments), sDCM accounts for endogenous or random fluctuations in hidden neuronal states that enable the analysis of resting state fMRI studies.^9,12^

In contrast to functional connectivity analyses—based upon correlations with a seed region or independent component analysis (ICA)—sDCM has a number of methodological and interpretational advantages. These include the ability to make inferences about directed and weighted (ie, excitatory or inhibitory) connections among neuronal sources. This is clearly important in terms of understanding cortical hierarchies and distributed processes, which are usually cast in terms of forward and backward connections. The disadvantages of sDCM are largely computational in nature, because the estimation (ie, model inversion) rests upon an iterative Bayesian inversion or filtering. This can take several minutes or even hours for multiple subjects.^9,12^

The purpose of the current study was to evaluate differences in effective connectivity, within the DMN, between patients with first-episode schizophrenia and healthy control subjects using sDCM. Based on recent formulations of the dysconnection hypothesis—in terms of predictive coding and hierarchical inference^13^—we postulated that afferents to the anterior frontal (AF) node of the DMN would show reduced effective connectivity. This follows from the notion that psychosis can be explained by an aberrant precision or confidence afforded to representations in the cortical hierarchy. In neuronally plausible implementations of predictive coding, precision is thought to be encoded by the postsynaptic sensitivity (ie, gain) of the external (ie, superficial) pyramidal layer (lamina III) cells of the cerebral neocortex reporting prediction errors.

In functional terms, hierarchical predictive coding casts recurrent message passing among different cortical areas as the transmission of ascending prediction errors and descending predictions (also known as top-down predictions or corollary discharges). Neural activity—at any level of the cortical hierarchy—is thought to encode expectations about the causes of a sensory input. These expectations generate top-down predictions that are compared with expectations at the level below. The resulting prediction error or mismatch is then passed forward to update expectations in the higher levels.^14–17^ The precision of, or confidence in, prediction error—at any level—determines how much that level constrains the expectations in the higher levels. Available evidence^18^ suggests that the primary deficit in schizophrenia may be a failure to attenuate precision at the lowest (sensory) cortical levels—leading to a failure of sensory attenuation and characteristic soft neurological signs (eg, abnormal pursuit eye movements).^18^ This primary deficit is assumed to induce compensatory increases in the precision of higher levels and consequent difficulties inferring the causes of sensations—causing, eg, hallucinations and delusions.^19^ This means that many of the symptoms and signs of schizophrenia can be understood as false perceptual inference, secondary to a failure of neuromodulation to optimize precision (cf, aberrant salience)^20^ at different levels of the cortical hierarchy. Therefore, we hypothesized a reduction in the effective connectivity of extrinsic (ie, between node) afferents to the hierarchically highest node of the DMN—the AF node—and an increase of its intrinsic (ie, within node) excitability. Put simply, we anticipated that the AF node would listen more to itself than to ascending messages from lower hierarchical levels. Associating the AF node with the highest level of the cortical hierarchy was based primarily on phylogenetic and ontogenetic arguments.^21^ In summary, we hypothesized a reduction in the effective connectivity of afferents to the AF node and a concomitant reduction of its recurrent self-inhibition.

## Methods

### Subjects

Between 2009 and 2011, 33 patients (9 women, mean age = 25.0 years, SD = 5) with the diagnosis of schizophrenia according to the Diagnostic and Statistic Manual of Mental Disorders, 4th edition, revised (DSM-IV-TR) criteria^22^ were prospectively included in this study. All patients fulfilled criteria for stage 2 (first episode of psychosis) of the staging classification system proposed by McGorry et al.^23^ The diagnosis of schizophrenia was established by consensus between 2 psychiatrists, according to the aforementioned criteria, at a specialized outpatient clinic for early detection of psychosis. We required all patients to have at least 4 years of education, and to be stable—clinically and pharmacologically—for at least 1 month prior to the fMRI scanning session. Patients fulfilling criteria for deficit schizophrenia were identified.^24^ Additional clinical assessment for patients included the Positive and Negative Syndrome Scale (PANSS) scores.^25^

Exclusion criteria comprised: acute infectious, neurological or tumoral pathology of the central nervous system, or other active pathology; history of significant head trauma; inability to provide written informed consent; illicit drug abuse or changes in psychopharmacological therapy (including type and dosage) during the month prior to scanning; cases of psychosis purely attributable to substance abuse; and patients with chronic schizophrenia.

First-episode and chronic schizophrenia were distinguished using a temporal cutoff of 5 years of illness. Given that cognitive impairment, gray matter loss, and social isolation are expected to be more prominent in patients with chronic schizophrenia, we hoped to suppress these potential confounds by only including patients with first-episode schizophrenia. In addition, given that patients with first-episode schizophrenia necessarily have a shorter exposure to antipsychotic drugs, we hoped to reduce the potentially confounding effects of medication.

Fifteen healthy control subjects (4 women, mean age = 24.6 years, SD = 4) matched for sex, age, and education (mean duration of education = 13.4 years, SD = 3) were included for comparison. History of illicit drug abuse and concurrent medication were taken into account—using the olanzapine dose equivalent^26^ for patients under antipsychotic therapy. The study was approved by the local ethics committee, and all subjects provided written informed consent.

### MRI Protocol

MRI data were acquired using a scanner operating at 3 Tesla (Trio A Tim, Siemens, Erlangen, Germany) and equipped with a 12-channel radiofrequency head coil. A blood oxygen level dependent (BOLD) fMRI time series (echo time [TE] = 25ms, repetition time [TR] = 3000ms, flip angle = 90°, field of view [FOV] = 192mm, slice thickness = 3mm, no inter-slice gap, number of repetitions = 260, acquisition matrix = 64 × 64, voxel resolution = 3 × 3 × 3mm, scanning time = 13 minutes) was acquired. Sagittal single-slab high-resolution three-dimensional magnetization-prepared rapid gradient echo (TE = 3ms, TR = 2300ms, flip angle = 9°, inversion time = 900ms, FOV = 240mm, slice thickness = 1.2mm, number of slices = 160, acquisition matrix = 256 × 256, voxel resolution = 1 × 1 × 1.2mm, scanning time = 9:14 minutes) T1-weighted images (T1-WI) were also acquired.

### BOLD fMRI Experiment

**Fig. 1.**
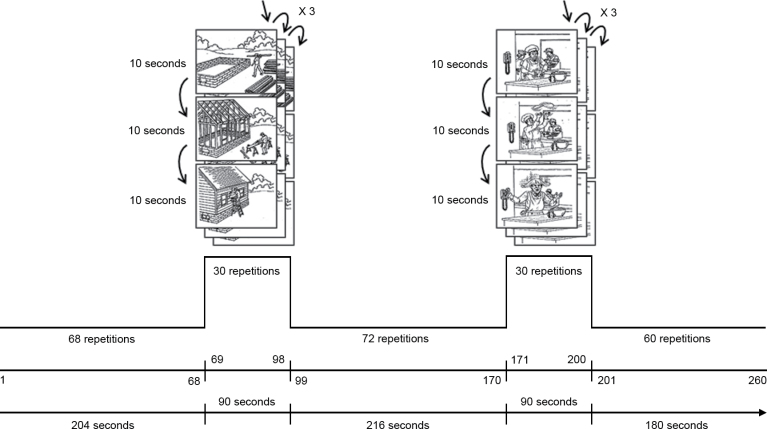
Blood oxygen level dependent functional magnetic resonance imaging experiment. Periods of resting state were interspersed with periods of visual stimulation using pictures extracted from the Picture Arrangement subtest of the Wechsler Adult Intelligence Scale-III.

The details and timing of our experiment are shown in figure 1. The predominantly resting state time series was interspersed with 2 extended periods of continuous picture viewing with drawings taken from the Picture Arrangement subtest of the Wechsler Adult Intelligence Scale-III (WAIS-III).^27^

The rationale for using an unusually long block design with a picture-viewing task paradigm, instead of a *pure* resting state experiment, was 2-fold. First, we were able to use the (within session) activation blocks to validate the DMN—based on endogenous fluctuations in the BOLD signal during rest periods—in terms of deactivation relative to picture viewing (this was confirmed,^28^ but it is not reported in the current article). Second, the long periods of picture viewing allowed us to characterize cognitive performance at the time of scanning. We used 2 extended periods of continuous picture viewing, with drawings taken from the Picture Arrangement subtest of the WAIS-III, because this subtest constitutes a measure of social cognition, and of ability to understand precursors and consequences of events. We used pictures with an intermediate degree of difficulty—asking the subjects 6 questions at the end of the fMRI scanning session and scoring the total number of correct answers. This allowed us to assess how well each subject understood the paradigm, as well as to assess working memory, attention and processing speed.

### Image and Statistical Analyses

We used the statistical parametrical mapping (SPM) software (http://www.fil.ion.ucl.ac.uk/spm/) for spatial processing. This included realignment, unwarping, unified segmentation of T1-WI, removal of non-brain voxels on segmented T1-WI, coregistration of fMRI time series to T1-WI, as well as normalization of functional and structural images to the standard Montreal Neurological Institute template at a spatial resolution of 1 × 1 × 1mm. In addition, fMRI images were smoothed with a full-width at half maximum Gaussian kernel of 6 × 6 × 6mm.

The 4 major nodes of the DMN were extracted from pre-processed fMRI time series of an independent set of 16 healthy volunteers (4 women, mean age = 24.6 years, SD = 5) matched for sex, age, and education of the control subjects. These subjects were exposed to the same fMRI experiment to ensure an unbiased, but context sensitive, identification of the DMN. We used group ICA of fMRI toolbox software (http://mialab.mrn.org/software/gift) to extract regional nodes as contiguous clusters over a *Z* score threshold of 2.5. Table 1 summarizes the spatial characteristics of the ensuing DMN nodes.

**Table 1.** Default Mode Network Nodes

	Number of Voxels	Centroid MNI Coordinates (mm)		
AF^a^	158	0	53	−2
PC	1215	0	−55	25
LP	484	−44	−66	31
RP	368	48	−62	31

*Note*: AF, anterior frontal; LP, left parietal; MNI, Montreal Neurological Institute; PC, posterior cingulate; RP, right parietal.

^a^The centroid of the AF node corresponds to a location in the cingulate sulcus. Therefore, the AF node comprises parts of both the anterior cingulate and medial prefrontal cortices.

To summarize the activity of these nodes, we used the principal eigenvariate of their constituent voxels in our independent patient and healthy control groups, following a standard SPM analysis of regional activity. This analysis used a general (ie, convolution) linear model based upon boxcar stimulus functions encoding the picture-viewing conditions. We also included the movement parameters from the realignment procedure as confounds and removed drift terms with a periodicity greater than 512 seconds. The adjusted time series from each of the DMN nodes, in each subject, were then used to create regional summaries.

These regional activities were modeled using sDCM, under a series of model architectures with an increasing number of connections. The best model was identified using random effects Bayesian model selection (BMS) based on the evidence for each model pooled over all subjects.^29^ This precluded any bias in subsequent tests for group differences in model parameters of effective connectivity. Random effects BMS is a hierarchical form of model selection that contrasts with a standard model selection, by allowing different models to be assigned to each subject and then evaluating the relative probability of different models over subjects.

**Fig. 2.**
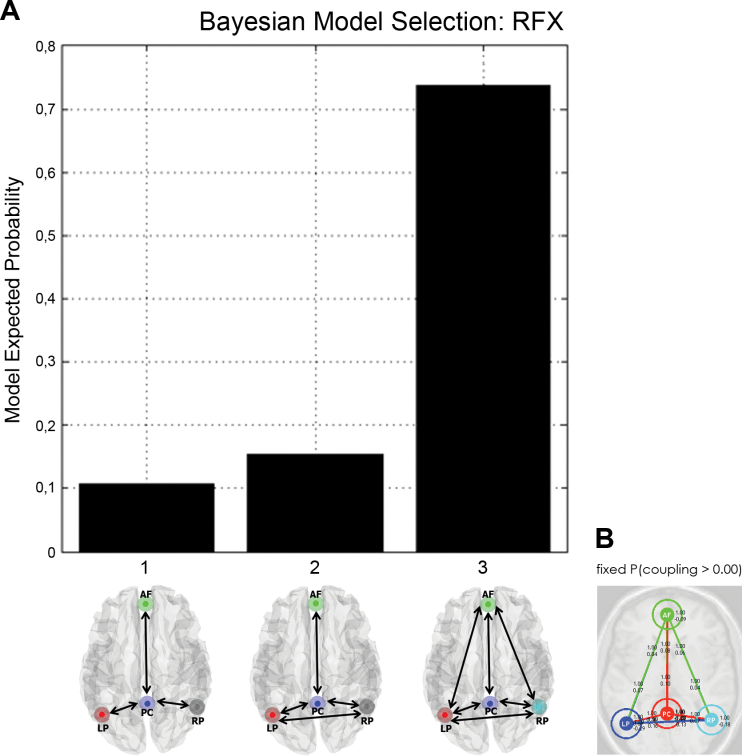
The fully connected (3) stochastic dynamic causal model (sDCM) was the best explanation, relative to simpler connectivity architectures (1 or 2), of the default mode network (A). Example of an sDCM model fit for one healthy subject (B). Probabilities of connections involving all nodes for this subject were = 1, and connection strengths (measured in Hz) were: PC-PC = −0.276; PC-AF = 0.076; PC-LP = 0.089; PC-RP = 0.068; AF-PC = 0.099; AF-AF = −0.092; AF-LP = 0.066; AF-RP = 0.041; LP-PC = 0.100; LP-AF = 0.044; LP-LP = −0.287; LP-RP = 0.128; RP-PC = 0.143; RP-AF = 0.056; RP-LP = 0.160; RP-RP = −0.176. AF, anterior frontal; LP, left parietal; PC, posterior cingulate; RP, right parietal.

BMS identified the fully connected model, in which the 12 possible extrinsic connections among the 4 nodes, and the 4 intrinsic connections in each node were included (figure 2).^30^ The fully connected model accommodated the effects of picture viewing as a set-related modulation of the extrinsic and intrinsic connections involving the PC and AF nodes. By definition, modulatory effects refer to condition-specific changes in effective connectivity (in this study, due to picture viewing). In other words, modulations model changes in the strength of effective connectivity in terms of user-specified free parameters of the dynamic causal model. Effective connectivity is measured in Hz, because the coupling is modeled as the rate of change of activity in one region caused by activity in another region. In short, effective connectivity in a dynamic causal model plays the role of a constant rate. We used SPM12a (revision 4729) for BMS and estimation of parameters (ie, effective connections and their modulation).

Although movement can cause coherent fluctuations in distributed signals that confound functional connectivity,^31,32^ effective connectivity estimates are relatively immune to head motion, because neuronal influences in DCM are mediated by correlations between signals and *changes in* signal—as opposed to correlations between signals per se. Nevertheless, we removed the effects of head motion at the within subject level—as previously described—by considering the realignment parameters as confounds when summarizing regional responses. At the between-subject level, a confound used to adjust for the effect of head motion—in subsequent comparisons—was quantified using the norm of the following matrix for each subject: 260 (length of fMRI BOLD time series) × 6 (realignment parameters).

Estimates of connection strengths were treated as summary statistics and used for classical inference about quantitative changes in connectivity between the groups, as well as for correlations with psychopathological scores within the patient group. Analyses of the summary statistics at the between-subject level were carried out with IBM SPSS 20.0 (www.ibm.com/software/analytics/spss/). These included the comparison of the strength of connections and their set-related modulations between patients and controls. Given that the corresponding values had an approximately normal distribution, we used the independent-samples Student’s *t* test to compare their means. Correlations between sDCM parameters and PANSS scores were tested using the Spearman’s rank correlation coefficient (*r*_*s*_). In addition, we used multiple linear regression analyses to determine whether sDCM parameters independently influenced psychopathology—after adjusting for the effects of age, sex, education, and head motion. We also used the Pearson’s chi-squared test to compare proportions of errors in responses to questions concerning the picture-viewing task paradigm. Statistical significance was considered when *P* values were <.05.

We report results of *t* tests at a level of significance uncorrected for the total number of connections compared, because our hypothesis was specifically about afferents to the AF node. Differences in all connections are reported, to illustrate the specificity of results. However, we adjusted the results for age, sex, education, history of illicit drug abuse, and head motion.

## Results

### Patient Sample

Table 2 summarizes the clinical characteristics of patients. On the basis of the DSM-IV-TR criteria, 28 (85%) of the 33 patients were diagnosed as having paranoid schizophrenia. Five (15%) patients fulfilled criteria for disorganized schizophrenia. Four of these fulfilled criteria for deficit schizophrenia. One-third (33%) of the patients had history of illicit drug abuse, including the past use of cannabis. Although the proportion of patients (46%) committing at least 1 error in responses to the picture-viewing questions was significantly higher (Pearson’s chi-square = 4.65; *P* < .05) than the corresponding proportion of healthy control subjects (13%), no subject made more than 2 errors. This indicates a generally good understanding of the pictures used and suggests a mild degree of cognitive impairment in the majority of patients on this paradigm, relative to healthy control subjects.

**Table 2.** Characteristics of Patients (*n* = 33) With First-Episode Schizophrenia, Including Age and Demographic Data, the Positive and Negative Syndrome Scale (PANSS) Scores, and Medication

Characteristic	Mean (SD)	Range
Sex	9 women (−)	—
Age	25.03 (4.5)	19–37
Education (y)	11.91 (3.0)	6–17
Duration of illness (mo)	15.10 (12.5)	0–40
PANSS positive^a^	12.19 (4.2)	7–23
PANSS negative^a^	18.96 (6.2)	8–31
PANSS general^a^	35.46 (10.2)	19–53
PANSS total^a^	66.62 (18.3)	35–97
Olanzapine dose equivalent (mg)	16.26 (6.6)	6.7–33.3

*Note*:^ a^Higher values indicate higher severity.

### sDCM Findings

**Fig. 3.**
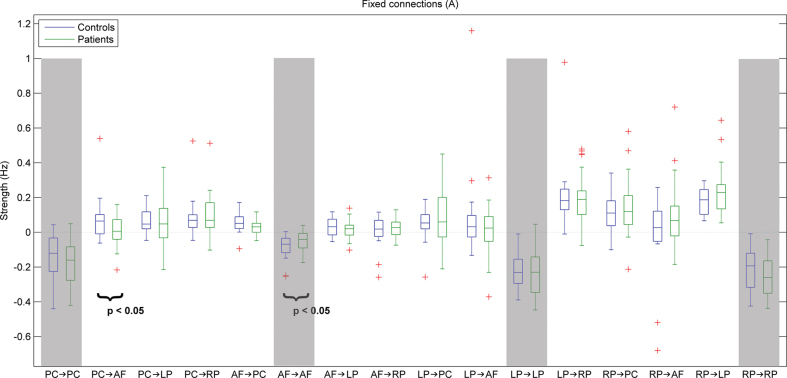
Box plots displaying strength of fixed connections (“A” values) between the default mode network nodes in controls (blue) and patients (green). These effective connectivity values are measured in Hz and correspond to rate constants (supplementary material). + symbol corresponds to outliers.

The overall profile of connectivity was remarkably consistent, both over subjects and between groups. This is important, because it speaks of the validity and efficiency of the DCM estimates. One can observe in figure 3 that nearly all subjects had connectivity estimates in the same direction and range. Furthermore, the profile of strengths over both groups was remarkably similar. This would not have happened if the estimates were inefficient (and, therefore, variable over subjects).

Despite the between group consistency, there were significant quantitative differences. As predicted, the strength of the directed connection from the PC to the AF node (PC-AF) of the DMN was significantly weaker in patients (mean value = 0.013 Hz, SD = 0.09) than in control subjects (mean value = 0.084 Hz, SD = 0.15; *P* < .05). In addition, the strength of the inhibitory intrinsic connection of AF (AF-AF) was significantly weaker in patients (mean value = −0.048 Hz, SD = 0.05) than in control subjects (mean value = −0.088 Hz, SD = 0.77; *P* < .05). After adjusting for the effects of age, sex, education, history of illicit drug abuse, and head motion, the associations between first-episode schizophrenia and weaker PC-AF or AF-AF connections remained significant (*P* < .05 and *P* < .01, respectively). This fits comfortably with the notion that the sensitivity of AF to ascending inputs is reduced in schizophrenia.

No other significant differences between patients and controls were detected with respect to the strength of any other connection. Modulatory effects were found to increase the excitatory extrinsic connections between PC and AF, as well as to increase the inhibitory intrinsic connections within these nodes, but no significant differences between patients and controls were detected.

### Associations Between sDCM Findings and Psychopathology

Significant correlations were found between the strength of the inhibitory self-connection of PC (PC-PC) and both the PANSS positive (*r*_*s*_ = .44; *P* < .05) and PANSS negative (*r*_*s*_ = .45; *P* < .05) scores. There was also a significant correlation between PC-PC and the PANSS total (*r*_*s*_ = .41; *P* < .05) score. In addition, the strength of connection from the PC node to the right parietal (RP) node (PC-RP) was found to be negatively correlated with the PANSS positive (*r*_*s*_ = .52; *P* < .01) score. No other significant correlations were found. After adjusting for the effects of age, sex, education, and head motion, only the association between PC-PC and the PANSS positive score remained statistically significant (*P* < .05).

## Discussion

Our results show weaker PC-AF extrinsic connectivity and reduced AF self-inhibition in the DMN of patients with first-episode schizophrenia, relative to healthy control subjects. In other words, patients with schizophrenia show a reduced sensitivity (ie, gain) to both extrinsic (ie, excitatory) and intrinsic (ie, recurrent inhibitory) afferents to the AF node. This is in agreement with theoretical accounts of the dysconnection hypothesis that appeal to predictive coding to explain false inference (eg, illusory deficits, hallucinations, and delusions) in schizophrenia.^18,33^ The results also confirm previous findings indicating connectivity reductions in schizophrenia,^34^ rather than increases,^6,35^ especially with respect to connections involving the frontal lobe.^34,35^ Moreover, they provide mechanistic insights into the way the PC and AF nodes of the DMN interact in schizophrenia.

The National Institute of Mental Health has recently launched the Research Domain Criteria (RDoC) project. The RDoC classification assumes that dysfunction in neural circuits underlies mental disorders, and that such dysfunction can be identified with clinical neuroscience tools, such as functional neuroimaging.^36^ Our results speak to this notion, confirming previous suggestions that dysfunction of the DMN in schizophrenia can be attributed to an altered interaction between the anterior cingulate and parietal cortices.^5^ Specifically, our results suggest that the excitatory influence of the PC node on the AF node of the DMN is, on average, more than 6 times weaker in patients with first-episode schizophrenia than in healthy control subjects, and that recurrent inhibitory influences of the AF node are likewise reduced to approximately 50% in patients. From the perspective of hierarchical predictive coding, these changes may reflect an aberrant precision or salience of prediction errors at high (prefrontal) levels of the cortical hierarchy. This aberrant precision can be understood—in simple terms—as a reduced confidence in (or attention to) ascending prediction errors that inform high level representations, such as concepts, memories, and plans. The implicit failure to encode precision can lead to false inference of the sort associated with many symptoms and signs of schizophrenia, as shown using computational simulations:^18^ According to Adams et al,^18^ the occurrence of hallucinations and delusions is seen as a compensatory increase in the precision or gain of the lamina III pyramidal cells, at high levels of the cortical hierarchy. This fits comfortably with the reduced self-inhibition of the AF node found in the current study. This also relates formally to a putative failure of corollary discharges in schizophrenia.^37^ As mentioned in the introduction, corollary discharges correspond to descending or top-down predictions.

The reduced influence of afferents to the AF cortex is also consistent with the neurochemical deficit of dopamine in the frontal lobe.^38^ Furthermore, it is consistent with neuropathological findings in schizophrenia, particularly with cytoarchitectural findings. These include reduction of synaptic density, predominantly affecting dendritic inputs to superficial pyramidal cells,^39^ the basis of the so-called “reduced neuropil hypothesis” of schizophrenia.^40^ This laminar-specific deficit is found in cortical association areas and the paralimbic cortex, including the prefrontal cortex.^41,42^ Crucially, many genes believed to confer risk of schizophrenia converge in the metabolism of the *N*-methyl-d-aspartate (NMDA) receptor of glutamate—one of the predominant molecular regulators of synaptic gain and plasticity—especially in NMDA-expressing synapses of the superficial (eg, lamina III) neocortical layer cells, but also through direct or indirect links to dopamine and gamma-aminobutyric acid signaling.^43^ This is important, because superficial neocortical layer cells have been implicated both theoretically and empirically in constructing predictions and prediction errors during perceptual synthesis and working memory. A key example here is the mismatch negativity that is consistently impaired in schizophrenia.^44–46^ In addition, mismatch negativity deficits are correlated with loss of grey matter in the frontal cortex.^47^ In short, there is converging evidence to suggest that a failure of gain control or neuromodulation—involving superficial pyramidal cells in the prefrontal cortex—may underlie the pathophysiology of schizophrenia. This failure of gain control is consistent with theoretical accounts of false inference from the perspective of predictive coding and hierarchical inference in the brain.^18,33,48^

A paradigm shift from schizophrenia as a single disease entity to a group of phenotypically similar diseases and syndromes has been increasingly acknowledged, although the current body of knowledge is still insufficient to disentangle their heterogeneity.^49,50^ Likewise, there is an incomplete understanding of their underlying etiology and pathophysiology.^51^ Our results illustrate how characterizing effective connectivity can elucidate pathophysiology—in what we currently diagnose as schizophrenia—and may help to differentiate patterns of abnormal connectivity of different subgroups of patients in the future.

Our analyses found a significant association between PC self-inhibition and increased severity of positive symptoms—after adjusting for the effects of other variables. This is intriguing, in relation to the main effect indicating a decreased self-inhibition of the AF node. It is especially interesting, in relation to the reported effects of psilocybin (a hallucinogenic drug) in a recent magnetoencephalography study showing desynchronization of the PC cortex.^52^ Curiously, the PC cortex has also been previously identified as an area of the DMN significantly more associated with positive symptoms in schizophrenia.^5^

It will be an interesting challenge to understand synaptic mechanisms in terms of hierarchical inference in the near future. The central role of neuromodulation and its functional encoding of precision may provide a promising lead. It should be noted that finding a significant correlation between effective connectivity and psychopathology lends the estimates of connectivity a predictive validity, which is reassuring, but more work is clearly needed to confirm the validity of (noninvasive) effective connectivity estimates of the sort.

Abnormalities in the neurodevelopmental process of myelination can result in altered temporal synchrony among different neural networks in schizophrenia,^53^ such as between the DMN and the so-called “salience network.”^8^ However, we did not assess effective connectivity between or within nodes of brain networks other than the DMN. Our hypothesis-led focus could be regarded as a limitation of the current study. The assessment of effective connectivity among nodes of brain networks other than the DMN may be usefully explored in future studies.

Another limitation of this study is its sample size. However, the fact that we were able to demonstrate significant differences between relatively small groups of subjects indicates that the effect sizes we report must be relatively large. We also acknowledge that the patients included in this study were receiving antipsychotic medication. This could have influenced the results. Nevertheless, it would have been difficult to include symptomatic patients, without medication, willing to cooperate with the fMRI scanning.

Our interpretation of the differences in directed connectivity can be regarded as somewhat speculative—resting upon assumptions about the neuronal implementation of predictive coding. However, the interpretation offered by predictive coding and aberrant precision fits comfortably with the aforementioned neurochemical and synaptic abnormalities in schizophrenia pointing to an abnormal control of cortical gain. In this context, the abnormal control of cortical gain can be considered as the pathophysiological counterpart of aberrant precision in predictive coding.

Clearly, DCM of fMRI cannot resolve the precise synaptic mechanisms of neuronal interactions, but the findings of selective abnormalities in the postsynaptic sensitivity (or gain control) of the prefrontal cortex in first-episode schizophrenia are entirely consistent with the deficits one would associate with aberrant precision or salience in predictive coding.

In conclusion, our DCM study suggests that the excitatory influence of the PC node on the AF node and the intrinsic inhibitory self-connection of the AF node are reduced in patients with first-episode schizophrenia. This is consistent with a synaptic dysconnection under current—predictive coding—formulations of false inference in schizophrenia, due to aberrant cortical gain control or precision.

## Supplementary Material

Supplementary material is available at http://schizophreniabulletin.oxfordjournals.org.

## Funding

This work was supported, in part, by Centro de Investigação Clínica (Hospital de São João), who granted the amount of 6098 euros to A.J.B.-L., C.S., A.N., and S.R. for a research project preceding the current article. G.R.R. was supported by the Medical Research Council (MR/J014257/1). K.J.F. and the Wellcome Trust Centre for Neuroimaging are supported by core funding from the Wellcome Trust (091593/Z/10/Z). The magnetic resonance imaging scanning sessions were granted by means of an agreement between the Department of Medical Imaging (Faculty of Medicine, University of Porto) and the Departments of Psychiatry and Radiology (Hospital de São João).

## Supplementary Material

Supplementary Data
