# Supplementary material for: Dysconnectivity Within the Default Mode in First-Episode Schizophrenia: A Stochastic Dynamic Causal Modeling Study With Functional Magnetic Resonance Imaging
Source: Schizophr Bull. 2014 Jun 17;41(1):144–53. doi: 10.1093/schbul/sbu080 (PMC4266292; doi:10.1093/schbul/sbu080)
Supplement: Supplementary Data [file supp_41_1_144__index.html]

Dysconnectivity Within the Default Mode in First-Episode Schizophrenia: A Stochastic Dynamic Causal Modeling Study With Functional Magnetic Resonance Imaging — Dysconnectivity Within the Default Mode in First-Episode Schizophrenia: A Stochastic Dynamic Causal Modeling Study With Functional Magnetic Resonance Imaging — Supplementary Data 

# Dysconnectivity Within the Default Mode in First-Episode Schizophrenia: A Stochastic Dynamic Causal Modeling Study With Functional Magnetic Resonance Imaging

## Supplementary Data

Data files

**Files in this Data Supplement:**

- Supplementary Data - Supplementary Data
