# Supplementary material for: Dysconnectivity Within the Default Mode in First-Episode Schizophrenia: A Stochastic Dynamic Causal Modeling Study With Functional Magnetic Resonance Imaging
Source: Schizophr Bull. 2014 Jun 17;41(1):144–53. doi: 10.1093/schbul/sbu080 (PMC4266292; doi:10.1093/schbul/sbu080)
Supplement: Supplementary Data [file supp_sbu080_2014_04_27_Supplementary_material.doc]

**Supplementary material**

**
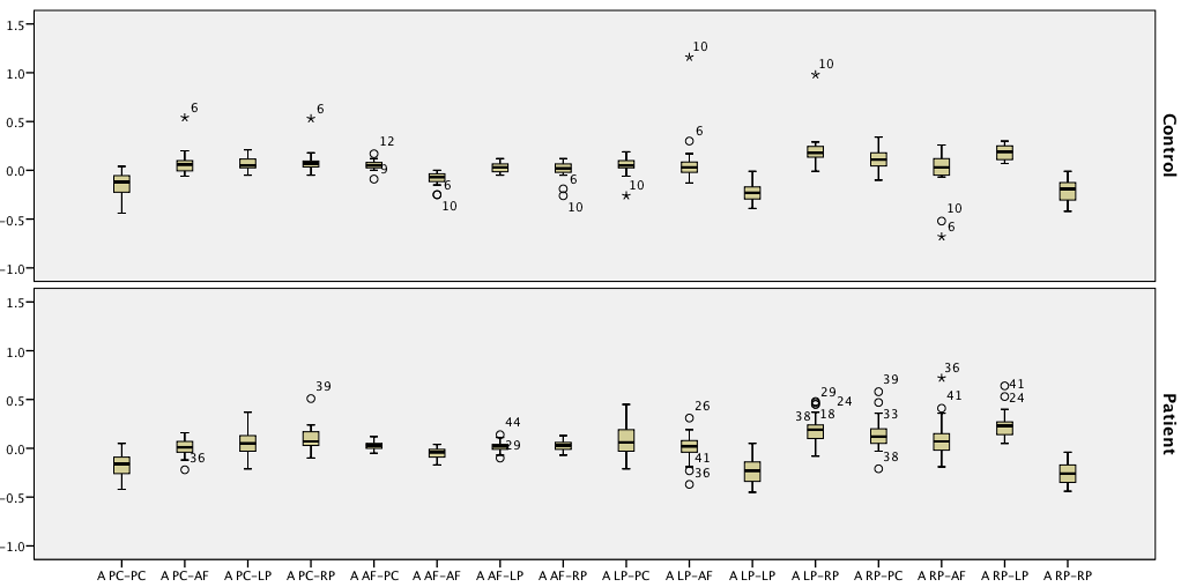
**

Box plots displaying strength of fixed connections (“A” values measured in Hz) between the default mode network nodes—discriminating outliers (circles) and extreme outliers (asterisks)—in controls (upper panel) and patients (lower panel). Note that the number of extreme outlier values is small, and that they are not consistent across connections.
